# Extraocular sebaceous carcinoma masquerading as eczema

**DOI:** 10.1093/skinhd/vzaf036

**Published:** 2025-07-30

**Authors:** Yu-Ting Tsai, Han-Chi Tseng

**Affiliations:** Department of Dermatology, Kaohsiung Chang Gung Memorial Hospital and Chang Gung University College of Medicine, Kaohsiung, Taiwan; Department of Dermatology, Kaohsiung Chang Gung Memorial Hospital and Chang Gung University College of Medicine, Kaohsiung, Taiwan; Graduate Institute of Clinical Medical Sciences, Chang Gung University, Taoyuan, Taiwan; Department of Dermatology, Kaohsiung Municipal Ta-Tung Hospital, Kaohsiung, Taiwan

## Abstract

A 49-year-old man presented with a slowly growing, pruritic erythematous scaly plaque on his left arm. Histopathology and immunohistochemistry confirmed extraocular sebaceous carcinoma (EOSC), and the patient underwent wide local excision with clear margins. This case highlights the importance of considering EOSC in the differential diagnosis of persistent eczematous lesions.

A 49-year-old otherwise healthy man presented with a slowly growing, pruritic lesion on his left arm of 5 years’ duration. He reported no systemic symptoms, including fever, malaise, unintentional weight loss or gastrointestinal issues, and denied any history of trauma or prior treatments. His family history was notable for his father’s death from oesophageal cancer at an advanced age. On examination, a 1.5 × 1 cm erythematous, scaly plaque was observed (Figure 1a). Dermoscopy revealed white scales on a red background (Figure 1b). There was no lymphadenopathy and no other skin lesions, including on the face. Histopathological analysis demonstrated lobules of neoplastic polygonal basaloid epithelial cells with irregular nuclei and focal bubbly cytoplasmic vacuolization (Figure 1c). Immunohistochemistry was positive for adipophilin (Figure 1d) and epithelial membrane antigen (Figure 1e), and negative for BerEP4, confirming the diagnosis of extraocular sebaceous carcinoma (EOSC). 18F-fluorodeoxyglucose positron emission tomography/computed tomography imaging showed no distant metastasis. The patient’s Mayo Muir-Torre syndrome risk score was 1, and additional immunohistochemical staining demonstrated proficient mismatch repair proteins. He subsequently underwent wide local excision with clear margins. Final staging was T1N0M0 (stage I) as per the 8th edition of the Union for International Cancer Control TNM classification. This case underscores the importance of considering EOSC in the differential diagnosis of persistent eczematous lesions.

**Figure 1:**
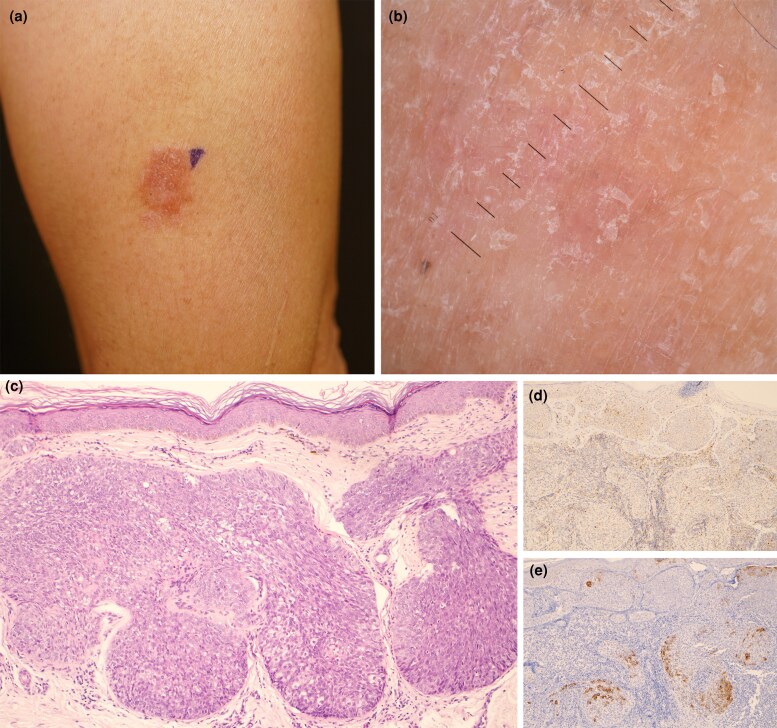
(a) An ill-defined erythematous, scaly plaque was on the left arm. (b) Dermoscopy revealed white scales diffusely distributed on a red background. (c) Skin biopsy showed lobules of neoplastic polygonal basaloid epithelial cells, bearing irregular nuclei and focal bubbly cytoplasmic vacuolization (haematoxylin and eosin stain ×100). Immunohistochemical staining confirmed the diagnosis of extraocular sebaceous carcinoma [(d) adipophilin ×100; (e) epithelial membrane antigen ×100].

## Data Availability

The data underlying this article will be shared on reasonable request to the corresponding author.

